# Changes in Attitudes and Beliefs Concerning Vaccination and Influenza Vaccines between the First and Second COVID-19 Pandemic Waves: A Longitudinal Study

**DOI:** 10.3390/vaccines9091016

**Published:** 2021-09-13

**Authors:** Alexander Domnich, Riccardo Grassi, Elettra Fallani, Alida Spurio, Bianca Bruzzone, Donatella Panatto, Barbara Marozzi, Maura Cambiaggi, Alessandro Vasco, Andrea Orsi, Giancarlo Icardi

**Affiliations:** 1 Hygiene Unit, San Martino Policlinico Hospital-IRCCS for Oncology and Neurosciences, 16132 Genoa, Italy; bianca.bruzzone@hsanmartino.it (B.B.); andrea.orsi@unige.it (A.O.); icardi@unige.it (G.I.); 2 SWG S.p.A., 34133 Trieste, Italy; riccardo.grassi@swg.it (R.G.); alida.spurio@swg.it (A.S.); 3 Seqirus S.R.L., 53035 Monteriggioni, Italy; elettra.fallani@seqirus.com (E.F.); maura.cambiaggi@seqirus.com (M.C.); alessandro.vasco@seqirus.com (A.V.); 4 Department of Life Sciences, University of Siena, 53100 Siena, Italy; 5 Department of Health Sciences (DISSAL), University of Genoa, 16132 Genoa, Italy; panatto@unige.it; 6 Faculty of Medicine and Surgery, University of Genoa, 16126 Genoa, Italy; s3509432@studenti.unige.it

**Keywords:** influenza, COVID-19, vaccination, vaccine hesitancy, attitudes, survey, Italy

## Abstract

Perceptions of the risks of vaccine-preventable diseases and preventive behaviors change over time. The ongoing COVID-19 pandemic may have modified laypeople’s attitudes towards routine vaccinations. In this longitudinal study, we aimed to assess changes in attitudes and beliefs concerning (influenza) vaccines between the first and second COVID-19 pandemic waves. A total of 1979 participants completed both 2020 and 2021 surveys. After one year, more interviewees agreed that vaccines were fundamental and should be mandatory (77.3% vs. 75.0%). Analogously, willingness to undergo influenza vaccination increased (*p* < 0.001) from 44.1% to 48.6%. This increase was seen in subjects aged ≥35 years. Previous influenza vaccinations, receipt of a COVID-19 vaccine, positive attitudes towards (influenza) vaccination, male sex, and older age were the main correlates of willingness to receive the 2021/22 influenza vaccine. Totals of 12.6% and 11.8% had no intention to receive the next seasonal influenza and COVID-19 vaccines, respectively. Most respondents favored a hypothetical combined influenza/COVID-19 vaccine (73.7%) or influenza and COVID-19 vaccine co-administration (67.5%). In Italy, influenza and COVID-19 vaccination hesitancy and refusal are common. Effective public health strategies to pursue higher uptake of both vaccines are urgently needed.

## 1. Introduction

Annual vaccination is a key public health intervention to reduce the socioeconomic burden of seasonal influenza. The World Health Organization (WHO) recommends [1] influenza vaccination (IV) for pregnant women, the elderly, children aged 6 months to 5 years, subjects with specific chronic conditions, and healthcare workers (HCWs). Despite these recommendations [1] and the well-known public health benefits [2,3], IV coverage rates are suboptimal in most at-risk groups and jurisdictions [4,5].

Italy was the first European country where SARS-CoV-2 spread significantly, with the so-called “first pandemic wave” starting in March 2020 [6]. Owing to fears of the possible co-circulation of SARS-CoV-2 and influenza viruses, which share several clinical signs and symptoms, some changes were made to Italian IV policy. Specifically, it was recommended that the start of the 2020/21 IV campaign should be brought forward to early October, and the free-of-charge vaccination offer was broadened to children aged 6 months to 6 years and older adults aged 60–64 years [7]. Some regions introduced compulsory IV for healthcare workers and/or the elderly (especially if institutionalized) [8,9,10]. The first available estimates of 2020/21 IV coverage rates suggest a significant increase in IV uptake among Italian HCWs [11], the general population, and the elderly [12].

In May 2020, we conducted a representative cross-sectional survey on attitudes and beliefs concerning seasonal IV [13]. Briefly, we found that most Italian adults judged IV positively, and that the main determinants of reluctance to receive the 2020/21 seasonal IV were younger age, lower perceived income, and no IV in the previous season. Moreover, about 20% of interviewees declared that, if no COVID-19 pandemic occurred, they would not receive the 2020/21 seasonal IV [13]. Since that time, several changes have occurred in the epidemiology of both influenza and SARS-CoV-2 infections and associated public health policies. First, the 2020/21 season in Europe saw a 99.8% reduction in influenza virus detections, with only 33 (of 25,606 specimens tested) positive samples reported by sentinel surveillance systems [14]. In Italy, no influenza virus detections were officially reported [15]. Second, since December 2020, alpha, beta, gamma, and delta variants of SARS-CoV-2 have been described [16]; these spread rapidly, owing to their greater transmissibility, becoming dominant in several countries [17,18]. Third, in December 2020, the first COVID-19 vaccines were granted conditional approval and a massive immunization campaign began [19].

These changes prompted us to transform an initial cross-sectional sample into a longitudinal panel in order to monitor public opinion towards (influenza) vaccination at different stages of the ongoing COVID-19 pandemic. In this paper, we describe changes in knowledge, attitudes, and beliefs regarding (influenza) vaccines between the first and second COVID-19 pandemic waves.

## 2. Materials and Methods

### 2.1. Study Design

The study population was composed of 2543 adults aged ≥18 years who participated in a cross-sectional online survey on 18–24 May 2020 with the aim of evaluating their beliefs, attitudes, and practices concerning (influenza) vaccination [13]. On 19–27 May 2021 (i.e., exactly one year after the first survey), participants were invited to take part in the second round of a computer-assisted web interview (CAWI).

The sample was representative of the Italian adult population from the point of view of principal socioeconomic characteristics, and was drawn from a pool of over 60,000 well-characterized individuals sampled by means of a two-stage probabilistic quota method. The sampling procedure is described elsewhere [13].

### 2.2. Questionnaire

In the present study, we slightly modified the original 2020 survey [13] by adding some novel items/response options that were judged essential in order to capture the above-described epidemiological and policy changes. However, most items were identical. Briefly, the first part recorded the participants’ principal socioeconomic characteristics: sex, age, macro-area of residence (North-East, North-West, Center, South and Islands), educational level, perceived income, and self-reported health status (excellent, very good, good, fair, and poor). The highest educational attainment reported was then converted to international standard classification of education (ISCED) levels, where level 1 corresponds to primary education [20].

The main variable of interest was willingness to receive the 2021/22 IV. Previous (seasons 2019/20 and 2020/21) IV uptake was also recorded in both surveys. Knowledge, attitudes, and beliefs concerning (influenza) vaccination were assessed through nine items rated on an anchored 4-point Likert-type response scale. Trust in different sources of information on IV (friends, traditional media, social networks, physicians, pharmacists, and public health institutions) was measured on a 1–10 scale, where 1 indicated the lowest level of trust. Those interviewees who were unlikely to receive the next seasonal IV were asked to indicate the main reasons for their decision.

Finally, participants were asked whether they had received/arranged/planned to receive any available COVID-19 vaccine and whether they would be willing to receive both influenza and COVID-19 vaccines at the same time or a combined influenza/COVID-19 vaccine.

The survey items are reported in Supplementary Table S1.

### 2.3. Statistical Analysis

Continuous and categorical variables were expressed as means with standard deviations (SDs) and percentages with 95% confidence intervals (CIs), respectively. Paired t and McNemar’s tests were used to compare repeated-measures continuous and dichotomous variables, respectively. The corresponding effect sizes were expressed as Cohen’s d and odds ratios (ORs), respectively.

Multivariable logistic regression analysis was performed in order to discern statistically significant associations between participants’ willingness to receive the 2021/22 seasonal IV and the above-described socioeconomic, vaccine-related, and attitudinal variables. The final model was selected by minimizing the Akaike information criterion (AIC). The independent variable “age” was treated as a continuous variable, since the different age categorization rules applied were associated in a substantially collinear manner with the variable “Employment pattern”. Possible multicollinearity issues in the final model were formally checked by quantifying the variance inflation factor (VIF) [21]. The explained variance was quantified by means of Nagelkerke’s pseudo-R^2^.

Statistical associations with a two-tailed α < 0.05 were deemed significant. Data analysis was performed by means of R stats packages, version 4.0.3 [22].

## 3. Results

### 3.1. Characteristics of the Study Panel

All 2543 individuals who participated in the first survey (2020) were invited to take part in this study. A total of 1981 agreed to do so (retention rate of 77.9%). At the time of the second survey (2021), two subjects (0.1%) no longer resided in Italy and were excluded from the analysis. Therefore, a total of 1979 paired 2020–2021 responses were analyzed. The mean age of participants was 48.3 (SD 15.1) years and males slightly prevailed (54.9%). Table 1 reports the principal socioeconomic and health-related characteristics of the study participants.

A total of 805 subjects (40.7%, 95% CI: 38.5–42.9%) had received at least one dose of a COVID-19 vaccine, while 357 (18.0%, 95% CI: 16.4–19.8%) stated that they had already booked their vaccination, and 584 (29.5%, 95% CI: 27.5–31.6%) claimed that they would do so as soon as possible. The remaining 233 subjects (11.8%, 95% CI: 10.4–13.3%) declared that they did not intend to be vaccinated against COVID-19.

### 3.2. One-Year Change in Knowledge, Attitudes, and Beliefs Concerning Vaccination and Influenza Vaccines

As in the 2020 survey, most participants judged (influenza) vaccination positively. However, there was a significant increase in trust in vaccinations in general. For instance, more people (77.3% vs. 75.0%) agreed that vaccines were crucial to public health and should be mandatory. Similarly, significantly fewer participants (18.3% vs. 25.6%) believed that vaccines were a “*fraud created only to enrich pharmaceutical companies*”. In 2021, more participants than in 2020 (82.6% vs. 78.9%) stated that they would like to have more information on vaccination. Regarding IVs, more people were aware of the different types available (59.7% vs. 51.8%) and their willingness to receive a more personalized IV increased (from 68.8% to 72.3%). Other attitudes towards IV did not change significantly (Table 2).

The newly introduced item “*All vaccines are safe*” produced the following output: 21.8% (95% CI: 20.0–23.7%), 46.6% (95% CI: 44.4–48.8%), 21.7% (95% CI: 19.9–23.6%), and 9.9% (95% CI: 8.6–11.3%) strongly agreed, more agreed than disagreed, more disagreed than agreed, and strongly disagreed, respectively.

In line with the previous survey, physicians, public health institutions, and pharmacists were believed to be the most trustworthy sources of information on IV. Generally, all information sources (except for friends) had higher average rankings in 2021. However, the effect size was small (d < 0.4). The largest increase in trust was seen with regard to traditional media and pharmacists (Table 3).

### 3.3. Influenza Vaccination in the Past Season, Willingness to Receive the 2021/22 Influenza Vaccination and Its Correlates

We first analyzed the actual 2020/21 IV uptake and compared it with the willingness to be immunized, as recorded in the 2020 survey. It emerged that most (84.9%) people who stated in 2020 that they would definitely have a flu shot actually did have a shot. Analogously, a total of 88.9% of respondents who had declared no intention to have a flu shot did not receive one. On the other hand, only 47.3% of interviewees who replied “Probably yes” were actually vaccinated (Table 4). Compared with subjects (N = 588) who had declared some willingness to receive IV and were actually vaccinated, those (N = 285) who were not vaccinated were significantly younger (44.6 vs. 56.7 years; *p* < 0.001); the effect size was large (d = 0.82).

**Figure 1:**
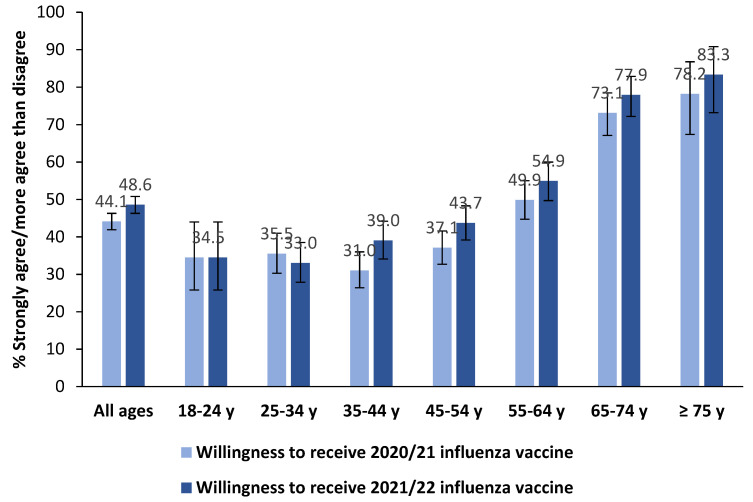
Between-survey comparison of the declared willingness ^a^ to receive 2020/21 and 2021/22 influenza vaccines, by age group (N = 1979). ^a^ Comprise response options “Strongly agree” and “More agree than disagree”.

Declared willingness to receive the seasonal IV significantly increased from 2020 to 2021 (from 44.1% to 48.6%, *p* < 0.001). This increase was seen in almost all age groups (except for young adults aged 18–34 years) (Figure 1). IV refusal was reported by 12.6% (95% CI: 11.2–14.2%) of respondents.

Notably, of those subjects who had already received, arranged for, or planned COVID-19 vaccination, a total of 42.2% (95% CI: 39.8–44.5%) declared that they had been vaccinated against influenza in the 2020/21 season. By contrast, only 10.3% (95% CI: 6.7–14.9%) of interviewees who declared no intention to be immunized against COVID-19 had received IV. This 4-fold difference was statistically significant (*p* < 0.001) and the effect size was large (OR 6.35; 95% CI: 4.12–9.78).

The main reasons for not having IV in the next season are reported in Table 5. Although the 2020 and 2021 surveys cannot be compared directly (since another response option was added to the 2021 survey), there was some decrease in participants’ selection of options regarding the effectiveness of IV. Indeed, in the 2021 survey, the most frequently selected (13.9%) option was “*Flu has diminished drastically since the COVID-19 pandemic began, so I don’t think a flu shot is necessary any more*”.

Finally, we investigated potential predictors of the likelihood of having the 2021/22 IV. As expected, the largest effect sizes were seen for previous IV vaccination in both the 2019/20 (aOR 4.11) and 2020/21 (aOR 13.62) seasons. Subjects already vaccinated against COVID-19 or those who planned to be so were also more prone to be vaccinated against influenza (aORs 5.52–9.46). Males and older people were more likely to have the intention to be vaccinated. Each 1-point increase in confidence that the respondent’s own physician is a reliable source of information on IV was associated with an 18% increase in the likelihood of having the 2021/22 IV. Moreover, several attitudes towards IV were significant predictors (Table 6). The model explained 65.5% of variance and no multi-collinearity issues emerged (VIFs < 1.4).

### 3.4. Public Opinion on the Co-Administration of COVID-19 and Influenza Vaccines and Willingness to Have a Combined Influenza/COVID-19 Vaccine

A total of 34.1% (95% CI: 32.0–36.2%) of respondents expressed firm willingness to receive both COVID-19 and IV at the same time, while 33.4% (95% CI: 31.3–35.5%) expressed some willingness to do so. Analogously, about three-quarters (73.7%, 95% CI: 71.7–75.6%) of interviewees favored having a combined influenza/COVID-19 vaccine. If such a combined vaccine were available, 34.8% (95% CI: 32.7–37.0%) stated that they “would definitely have it”, and 35.9% (95% CI: 33.8–38.1%) replied “I think I would have it”.

## 4. Discussion

This longitudinal study revealed a significant 1-year increase in respondents’ overall confidence in vaccines and willingness to receive the next seasonal IV. Some socioeconomic and attitudinal factors were independently associated with the propensity to being immunized in the upcoming 2021/22 season. We will now discuss the principal findings in the context of the available Italian and international research and provide suggestions for future IV campaigns.

Both influenza-associated risk perception and preventive behaviors may change over time. This dynamic pattern was, for example, reported during the last A(H1N1)pdm09 pandemic [23] and the ongoing COVID-19 pandemic [24]. Indeed, we ascertained a 10% relative increase (from 44.1% in 2020 to 48.6% in 2021 surveys) in people’s intention to be immunized against influenza. It is, however, unclear whether this growth in willingness will translate into increased coverage. On the other hand, in our surveys, we observed a 45.0% relative increase (from 2019/20 to 2020/21) in self-declared IV uptake, which is very close to the officially reported [12] increase of 41.1% (from 16.8% to 23.7%) in IV coverage in the general population.

Only a few studies on changes in attitudes and beliefs concerning IV in the COVID-19 era are currently available. The Vaccine Confidence Project [25] reported an increase (from 2018 to 2020) in public trust in vaccines, and in IVs in particular, across most European countries. In Italy, overall vaccine confidence grew from 53% to 60%, while public awareness that IV is important rose from 67.6% to 78.4%. A similar significant increase was seen in other European countries, with the exceptions of Estonia, Romania, Hungary, and the United Kingdom, where no significant changes were recorded. Among other possible reasons, the authors linked this increase to the perceived severity of the COVID-19 pandemic [25]. An Italian study by Caserotti et al. [24] reported an increase in willingness to receive IV from the pre-lockdown period (from the end of February to March 8, 2020) to the post-lockdown period (from May 19 to the end of June 2020) (aOR 1.82; 95% CI: 1.16–2.87). By contrast, a six-wave longitudinal study conducted in California between March and August 2020 showed a decreasing trend in intentions to receive IV [26]. These apparent discrepancies may be explained by several factors, including different survey time periods, and therefore SARS-CoV-2 epidemiology and restrictive public health measures adopted by single jurisdictions. In Italy, the observed increase in intentions to receive IV may also be explained by the “more effective” influenza prevention campaign promoted by the Ministry of Health in 2020 [27].

According to the effect size observed, the main drivers of the uptake of the next seasonal IV were both previous IV vaccination and COVID-19 vaccination status (or at least intention to be vaccinated against COVID-19). Indeed, IV in the previous season has been systematically shown to determine current IV status in all principal risk groups, including HCWs, the elderly, pregnant women, young children, and subjects with underlying health conditions [28,29,30,31,32]. However, we noted that, although IV status in both previous seasons (2019/20 and 2020/21) was a positive predictor of the next IV, the effect size of the latter was about three times higher than that of the former. In an Italian study by Fabiani et al. [33], elderly subjects vaccinated in the two previous seasons were 8.36 (95% CI: 8.17–8.55) times more likely to receive the 2016/17 IV than those who had not been vaccinated. Indeed, if people have a positive initial experience, most of them will probably seek IV the following year [29]. It is therefore essential to ensure the continuity of effective IV counseling strategies. In this regard, the role of general practitioners, who are the most trusted source of information on IV, is central.

Unwillingness to receive IV and COVID-19 vaccines may be interrelated. In a UK survey, willingness to undergo COVID-19 vaccination was significantly associated with willingness to receive a 2020/21 IV [34]. Analogously, among Italian undergraduates, previous IV was associated with greater acceptance of COVID-19 vaccination (aOR 3.81; 95% CI: 1.18–12.27) [35]. Finally, a meta-analysis of four studies showed that previous IV was a strong predictor (OR 3.17; 95% CI: 1.84–5.46) of COVID-19 vaccine acceptance [36]. It is therefore likely that effective vaccination counseling on one infection may have indirect positive effects on the other.

Of the structural social determinants analyzed, only increasing age and male sex were associated with a greater intention to receive the 2021/22 IV. That older age is a correlate of IV acceptance has been amply demonstrated by a number of systematic reviews and/or meta-analyses [28,29,30,31]. By contrast, the role of sex remains to be clarified. Although men tend to report a higher uptake rate than women, the difference becomes non-significant on adjusted analysis [29]. It is also possible that there is a significant interaction between age and sex; it has been shown that IV uptake in females decreases with increasing age, but increases in males [37]. In our model, the interaction term was not statistically significant.

In our study, positive attitudes towards IV were associated with greater willingness to receive the next seasonal IV. This finding is in line with those of a systematic review by Schmid et al. [28], which reported that a negative attitude towards IV was a major barrier to IV uptake in all principal risk groups. Among the various negative attitudes analyzed, lack of confidence in the efficacy of IVs held a prominent place [28]. A recent US survey [38] has shown a significantly reduced probability of taking the COVID-19 vaccine if the vaccine effectiveness was 50% (comparison to 70% or 90%), while the difference between a 70% and a 90% protection rate was not statistically significant. Laypeople’s statistical literacy is generally low [39]. Indeed, Tentori et al. [40] reported that most people are unaware of the meaning of vaccine effectiveness and confuse this term with the non-incidence rate among vaccinated people. This misinterpretation leads the overall undervaluation of the individual benefits of the vaccine. Moreover, according to the authors, this misunderstanding was aligned with expectations based on misreports in the media [40]. It has been reported [41] that the media often report unbalanced messages from the point of view of completeness, transparency, and correctness. We therefore believe that the media should convey their messages through a more balanced reporting and, ideally, adopt shared standards and norms. In our study, the traditional media (e.g., TV or newspapers) showed the largest 1-year increase as a trusted information source. In this regard, interventions such as media training for medical experts and regular meetings that may facilitate communication between experts and journalists may be beneficial [42].

More generally, it is still unclear whether the expected vaccine effectiveness should be provided to laypeople. Zhao et al. [43] have stated that “*vaccine effectiveness studies are designed to inform public health decisions rather than for individual decision-making*” and “*an individual’s decision to get vaccinated should be primarily informed by their risk of influenza illness and their risk of transmitting influenza to vulnerable people*”. We, however, believe that anticipated suboptimal (which varies substantially by season, location, age group, and average 40–60% [44]) IV effectiveness should be openly disclosed by healthcare providers. In fact, even if the efficacy is only 20% (and the coverage rate is 43%), IV is still able to avert 20.99 million infections, 129,701 hospitalizations, 61,812 deaths, and 2.22 million disability-adjusted life years [45]. Effective public health communication strategies should therefore provide balanced and laypeople-friendly risk–benefit information on immunization. In turn, effective vaccine promotion campaigns should stress the importance of talking to a healthcare professional about all vaccination aspects, including safety and effectiveness [46].

For what concerns the main reasons for not having a flu shot, our 2021 survey revealed a substantial 1-year decrease in respondents’ selection of options regarding the presumed low effectiveness of IV. On the other hand, the leading reason for not having a flu shot in the upcoming 2021/22 season was the apparent disappearance of influenza viruses in the previous 2020/21 season [15]. The epidemiology of influenza and influenza-like illness in the upcoming 2021/22 season is unclear and will probably depend on non-specific COVID-19 pandemic prevention measures, such as social distancing, lockdowns, the wearing of masks, etc. Several scenarios of the evolution of influenza in the COVID-19 pandemic era (e.g., influenza viruses will return, and the same clades will circulate; influenza viruses will return, but some subtypes/lineages/clades will disappear; influenza viruses will return, causing occasional outbreaks) have been proposed [47]. Moreover, by exerting the so-called “trained immunity” effect (i.e., by boosting the innate immune system), IV may reduce the incidence of some COVID-19-related outcomes. A systematic review and meta-analysis by Wang et al. [48] recently demonstrated a 14% (aOR 0.86, 95% CI: 0.81–0.91) reduction in the odds of being positive to SARS-CoV-2 in subjects vaccinated against influenza, as compared with non-vaccinated subjects. This argument could also be used to increase IV acceptance. It is clear that influenza (at least type A), being a zoonosis, cannot be eliminated; IV uptake goals [1] must therefore be pursued.

Over one year, people’s awareness of the existence of different IVs increased. In our opinion, two main factors may have contributed to this increase. First, ongoing controversies over the effectiveness and safety of different COVID-19 vaccines [49] may have increased people’s willingness to receive one vaccine type rather than another. Second, in 2020, four novel influenza vaccines were authorized and/or commercialized in Italy: quadrivalent egg-based standard-dose adjuvanted, quadrivalent egg-based high-dose, quadrivalent recombinant, and quadrivalent live-attenuated vaccines [50].

Finally, most interviewees looked favorably on the idea of receiving both IV and COVID-19 shots at the same time and/or a combined influenza/COVID-19 vaccine. From the public health perspective, vaccine co-administration or combined vaccines have several benefits, including fewer missed opportunities to vaccinate, simplified immunization schedules, logistical advantages, and reduced costs [51,52]. The first available clinical data [53] suggest that influenza and COVID-19 vaccines can be co-administered with little interference in terms of immunogenicity and efficacy and only a slight increase in solicited adverse events. By contrast, the rate of unsolicited adverse events was similar among the study arms. The most recent interim clinical considerations for the use of COVID-19 vaccines issued by the Centers for Disease Control and Prevention (CDC) [54] suggest that SARS-CoV-2 and other vaccines may be co-administered without regard to timing, but each shot should be inoculated in a different injection site. Further large-scale phase III and pharmacovigilance studies on vaccine co-administration (in terms of both immunological inference and safety) are warranted.

Analogously, the first preclinical studies suggest that combined vaccines are promising. A combination of a Matrix-M-adjuvanted quadrivalent nanoparticle IV and NVX-CoV2373 COVID-19 vaccine was immunogenic and efficacious in ferret and hamster models [55]. Another approach consists of a recombinant influenza type A virus genetic platform that encodes the receptor-binding domain of SARS-CoV-2. This vaccine candidate also proved immunogenic and efficacious against lethal challenge by both viruses [56].

Our study has some limitations, which should be considered when interpreting the results. First, like all web-based surveys, our study may have been subject to the digital divide bias. However, both probabilistic quota sampling and the longitudinal nature of the survey should mitigate the effects of this bias. Second, self-reported IV uptake was substantially higher than that officially registered. For instance, in this study, self-reported 2019/20 IV uptake (results not shown) was 18.1% (95% CI: 15.5–20.9%), 23.7% (95% CI: 20.9–26.7%), and 45.6% (95% CI: 40.1–51.1%) among subjects aged 18–44, 45–64, and ≥65 years, respectively. The corresponding officially reported [57] statistics were 3.1%, 9.6%, and 54.6%, respectively. This discordant result is unlikely to have been due to the characteristics of the sample (representative of the adult Italian population) or participation bias (interviewees were not aware of the survey topic beforehand). Possible reasons include the social desirability [58] and recall (especially in subjects with irregular IV uptake patterns) [59] biases.

## 5. Conclusions

In conclusion, although a significant proportion of Italian adults are reluctant/hesitant toward both influenza and COVID-19 vaccines, public confidence in (influenza) vaccines increased significantly. This positive trend was at least partially determined by the ongoing COVID-19 pandemic. Our future work will focus on the continuous (at least two surveys per year) follow-up of the same panel, in order to capture even small changes in laypeople’s knowledge, attitudes, and beliefs concerning influenza vaccination. Moreover, future research should scrutinize laypeople’s attitudes towards safety aspects.

## Figures and Tables

**Table 1:** Socioeconomic characteristics of the study population (N = 1979).

Variable	Level	% (N)	95% CI
Sex	Male	54.9 (1086)	52.6–57.1
	Female	45.1 (893)	42.9–47.3
Age group, years	18–24	5.7 (113)	4.7–6.8
	24–34	16.2 (321)	14.6–17.9
	35–44	18.9 (374)	17.2–20.7
	45–54	23.7 (469)	21.8–25.6
	55–64	18.9 (375)	17.2–20.7
	65–74	12.6 (249)	11.2–14.1
	≥75	3.9 (78)	3.1–4.9
Geographic area	North-East	19.0 (376)	17.3–20.8
	North-West	28.0 (555)	26.1–30.1
	Center	21.2 (419)	19.4–23.0
	South	21.9 (434)	20.1–23.8
	Islands	9.9 (195)	8.6–11.3
ISCED educational level	1	0.7 (14)	0.4–1.2
	2	7.8 (154)	6.6–9.1
	3–4	48.0 (949)	45.7–50.2
	5	41.5 (821)	39.3–43.7
	6	2.1 (41)	1.5–2.8
Employment pattern	Employed	63.7 (1261)	61.6–65.8
	Student	6.2 (122)	5.1–7.3
	Housekeeper	6.1 (121)	5.1–7.3
	Unemployed	5.8 (114)	4.8–6.9
	Retired	16.1 (319)	14.5–17.8
	Other/prefer not to reply	2.1 (42)	1.5–2.9
Perceived income	Low	2.0 (39)	1.4–2.7
	Lower than average	7.6 (150)	6.5–8.8
	Average	32.3 (639)	30.2–34.4
	Higher than average	42.7 (846)	40.6–45.0
	High	2.0 (40)	1.5–2.7
	No personal income	13.4 (265)	11.9–15.0
Self-reported health status	Excellent	9.1 (181)	7.9–10.5
	Very good	45.5 (901)	43.3–47.8
	Good	42.0 (832)	39.9–44.3
	Fair	3.0 (59)	2.3–3.8
	Poor	0.3 (6)	0.1–0.7
Influenza vaccination in 2019/20 season	Yes	26.5 (524)	24.5–28.5
	No	73.5 (1455)	71.5–75.5

ISCED: international standard classification of education.

**Table 2:** Responses on knowledge, beliefs, and practices regarding (influenza) vaccination, by survey (N = 1979).

Item	% (95% CI)				*p*
	Agreed ^a^ in Both 2020 and 2021	Agreed ^a^ in 2021 But Disagreed ^b^ in 2020	Agreed ^a^ in 2020 But Disagreed ^b^ in 2021	Disagreed ^b^ in Both 2020 and 2021	
Vaccines are crucial to guaranteeing public health and should be mandatory	64.9 (62.8–67.0)	12.3 (10.9–13.9)	10.1 (8.8–11.5)	12.7 (11.2–14.2)	0.037
I need more information on vaccines	69.1 (67.0–71.1)	13.5 (12.1–15.1)	9.8 (8.5–11.2)	7.6 (6.5–8.8)	0.001
Vaccines are a fraud designed to profit pharmaceutical companies	12.0 (10.6–13.5)	6.3 (5.3–7.5)	13.5 (12.1–15.1)	68.1 (66.0–70.2)	<0.001
Influenza vaccination is a human right and must be guaranteed for people that would like to be vaccinated	82.5 (80.8–84.2)	7.5 (6.4–8.7)	7.1 (6.0–8.3)	2.9 (2.2–3.7)	0.72
It is unacceptable that there are no influenza vaccines in the coming season for people that would like to be vaccinated	77.7 (75.8–79.5)	9.5 (8.2–10.9)	8.4 (7.3–9.8)	4.4 (3.5–5.4)	0.29
If there were no free-of-charge influenza vaccine, I would pay for it out of my own pocket	38.2 (36.0–40.3)	15.4 (13.8–17.1)	14.4 (12.8–16.0)	32.1 (30.0–34.2)	0.41
There are different types of influenza vaccine	37.0 (34.9–39.2)	22.7 (20.9–24.7)	14.8 (13.3–16.4)	25.5 (23.6–27.4)	<0.001
I would be more willing to receive a flu shot if it were personalized	56.5 (54.3–58.7)	15.8 (14.2–17.4)	12.2 (10.8–13.8)	15.5 (13.9–17.1)	0.003

^a^ Comprise response options “Strongly agree” and “More agree than disagree”; ^b^ Comprise response options “Strongly disagree” and “More disagree than agree”.

**Table 3:** One-year change in perceived credibility of different sources of information on influenza vaccination, by survey (N = 1979).

Information Source	Mean (SD)		*p*	Effect Size, d
	2020 Survey	2021 Survey		
My physician	7.4 (2.2)	7.9 (2.0)	<0.001	0.23
Public health institutions	6.8 (2.4)	7.3 (2.2)	<0.001	0.22
My pharmacist	6.3 (2.3)	7.0 (2.1)	<0.001	0.32
Newspapers/TV	4.6 (2.2)	5.5 (2.2)	<0.001	0.37
Friends	4.4 (2.3)	4.5 (2.3)	0.054	0.04
Social media	3.2 (2.3)	3.7 (23)	<0.001	0.22

**Table 4:** Between-survey comparison of the declared willingness to receive the 2020/21 influenza vaccine (2020 survey) and actual 2020/21 vaccine receipt.

Willingness to Receive 2020/21 Influenza Vaccine (2020 Survey)	2020/21 Season Reported Vaccination (2021 Survey), % (95% CI)	
	Yes	No
Definitely yes (N = 465)	84.9 (81.4–88.1)	15.1 (11.9–18.6)
Probably yes (N = 408)	47.3 (42.4–52.3)	52.7 (47.7–57.6)
I do not know (N = 282)	18.1 (13.8–23.1)	81.9 (76.9–86.2)
Probably not (N = 444)	17.8 (14.3–21.7)	82.2 (78.3–85.7)
Definitely not (N = 380)	11.1 (8.1–14.6)	88.9 (85.4–91.9)
Total (N = 1979)	38.4 (36.3–40.6)	61.6 (59.4–63.7)

**Table 5:** Reasons for not having influenza vaccination, by survey.

Reason	2020 (N = 997)	2021 (N = 887)
Influenza vaccines are designed only to profit pharmaceutical companies	21.0 (18.5–23.6)	12.9 (10.7–15.2)
Influenza vaccines do not work	17.9 (15.5–20.4)	12.0 (9.9–14.3)
I am afraid of needles	8.5 (6.9–10.4)	8.2 (6.5–10.2)
I had a flu shot but developed a fever/cold anyway	8.5 (6.9–10.4)	5.5 (4.1–7.2)
My doctor advised against it	7.9 (6.3–9.8)	7.0 (5.4–8.9)
Flu has diminished drastically since the COVID-19 pandemic began, so I do not think a flu shot is necessary any more	NA ^a^	13.9 (11.7–16.3)
Other	36.2 (33.2–39.3)	40.6 (37.3–43.9)

^a^ This item was not assessed in the 2020 survey.

**Table 6:** Multivariable logistic regression analysis to predict willingness to receive the 2021/22 influenza vaccine.

Variable	Level	aOR (95% CI)	*p*
Sex	Female	Ref	-
	Male	1.34 (1.03–1.75)	0.032
Age	1-year increase	1.01 (1.00–1.02)	0.014
Influenza vaccination in the 2019/20 season	No	Ref	-
	Yes	4.11 (2.73–6.19)	<0.001
Influenza vaccination in the 2020/21 season	No	Ref	-
	Yes	13.62 (9.64–19.25)	<0.001
COVID-19 vaccination status	No, and I do not intend to receive a shot in the future	Ref	-
	Not yet, but I will receive a shot as soon as possible	5.52 (2.60–11.69)	<0.001
	Not yet, but I have already booked my shot	6.13 (2.85–13.21)	<0.001
	Yes	9.46 (4.48–19.97)	<0.001
Vaccines are crucial to guaranteeing public health and should be mandatory	Disagree ^a^	Ref	-
	Agree ^b^	1.87 (1.25–2.80)	0.002
All vaccines are safe	Disagree ^a^	Ref	-
	Agree ^b^	1.53 (1.10–2.14)	0.011
Influenza vaccination is a human right and must be guaranteed	Disagree ^a^	Ref	-
	Agree ^b^	2.08 (1.16–3.73)	0.014
It is unacceptable that there are no influenza vaccines in the future season	Disagree ^a^	Ref	-
	Agree ^b^	2.83 (1.63–4.89)	<0.001
If there were no free-of-charge influenza vaccine, I would pay for it out of my own pocket	Disagree ^a^	Ref	-
	Agree ^b^	1.64 (1.25–2.16)	<0.001
I would be more inclined to receive a flu shot if it were personalized	Disagree ^a^	Ref	-
	Agree ^b^	2.37 (1.69–3.34)	<0.001
Confidence in one’s own physician	1-point increase	1.18 (1.09–1.28)	<0.001

^a^ Comprise response options “Strongly disagree” and “More disagree than agree”; ^b^ Comprise response options “Strongly agree” and “More agree than disagree”.

## Data Availability

All relevant data are within the article.

## Supplementary Materials

The following are available online at https://www.mdpi.com/article/10.3390/vaccines9091016/s1, Table S1: Survey Instrument.
