# Supplementary material for: Changes in Attitudes and Beliefs Concerning Vaccination and Influenza Vaccines between the First and Second COVID-19 Pandemic Waves: A Longitudinal Study
Source: Vaccines (Basel). 2021 Sep 13;9(9):1016. doi: 10.3390/vaccines9091016 (PMC8470379; doi:10.3390/vaccines9091016)
Supplement: Supplementary file 1 [file vaccines-09-01016-s001.zip › vaccines-1349309-supplementary.pdf]

Table S1. Survey Instrument.

Item	2020 survey	2021 survey
Did you have a flu shot in the last season?<br>- I have never had a flu shot;<br>- I had a flu shot in the past, but not in the last season;<br>- I had a flu shot in the last season for the first time;<br>- I had a flu shot both in the last season and sometimes in the past.	Yes	Yes
To what extent do you agree or disagree with the following statement?<br>Vaccines are a fraud designed to profit the pharmaceutical companies.<br>- Strongly agree;<br>- More agree than disagree;<br>- More disagree than agree;<br>- Strongly disagree.	Yes	Yes
To what extent do you agree or disagree with the following statement?<br>Vaccines are crucial to guaranteeing public health and should be mandatory.<br>- Strongly agree;<br>- More agree than disagree;<br>- More disagree than agree;<br>- Strongly disagree.	Yes	Yes
To what extent do you agree or disagree with the following statement?<br>All vaccines are safe.<br>- Strongly agree;<br>- More agree than disagree;<br>- More disagree than agree;<br>- Strongly disagree.	No	Yes
To what extent do you agree or disagree with the following statement?<br>I need more information on vaccines.<br>- Strongly agree;<br>- More agree than disagree;<br>- More disagree than agree;<br>- Strongly disagree.	Yes	Yes
To what extent do you agree or disagree with the following statement?<br>Influenza vaccination is a human right and must be guaranteed for people that would like to have it.<br>- Strongly agree;<br>- More agree than disagree;<br>- More disagree than agree;<br>- Strongly disagree.	Yes	Yes
To what extent do you agree or disagree with the following statement?<br>It is unacceptable that there are no influenza vaccines in the future season for people that would like to be vaccinated.<br>- Strongly agree;<br>- More agree than disagree;<br>- More disagree than agree;<br>- Strongly disagree.	Yes	Yes
To what extent do you agree or disagree with the following statement?<br>If there were no a free-of-charge influenza vaccine, I would pay for it out of my own pocket.<br>- Strongly agree;<br>- More agree than disagree;<br>- More disagree than agree;<br>- Strongly disagree.	Yes	Yes
To what extent do you agree or disagree with the following statement?<br>There are different influenza vaccine types.<br>- Strongly agree;<br>- More agree than disagree;<br>- More disagree than agree;<br>- Strongly disagree.	Yes	Yes
To what extent do you agree or disagree with the following statement?<br>I would be more willing to get a flu shot if it were personalized.<br>- Strongly agree;<br>- More agree than disagree;	Yes	Yes

- More disagree than agree;<br>- Strongly disagree.		
Regarding influenza vaccination, on a scale from 1 (not at all) to 10 (completely) how much do you trust information from each of the following sources?<br>- Friends and acquaintances;<br>- My physician;<br>- My pharmacist;<br>- Public health institutions;<br>- TV/newspapers;<br>- Social networks.	Yes	Yes
Do you intend to have a flu shot in the upcoming season?<br>- Yes definitely;<br>- Probably yes;<br>- I don't know;<br>- Probably not;<br>- Definitely not.	Yes	Yes
What are the main reasons why you would not get a flu shot? Select up to 2 of the following:<br>- Influenza vaccines do not work;<br>- I had a flu shot but got a fever/cold anyway;<br>- I'm afraid of needles;<br>- Influenza vaccines are designed only to profit the pharmaceutical companies;<br>- My doctor advised against it;<br>- Flu has diminished drastically since the COVID-19 pandemic began, so I don't think it is necessary any more;[a]<br>- Other.	Yes	Yes
If there were an influenza vaccine shortage during the next season, whose fault it would be? Select up to 2 of the following:<br>- Ministry of Health;<br>- Pharmaceutical companies;<br>- Regional authorities;<br>- Local health units;<br>- Pharmacies;<br>- Wholesalers;[a]<br>- Other.	Yes	Yes
Have you been vaccinated against COVID-19?<br>- Yes;<br>- Not yet, but I have already booked my shot;<br>- Not yet, but I will get a shot as soon as possible;<br>- No, and I don't intend to get a shot in the future.	No	Yes
If it were possible, would you have both the COVID-19 and flu shots at the same time?<br>- Totally agree;<br>- Agree;<br>- Disagree;<br>- Totally disagree.	No	Yes
What do you think of the idea of having a combined COVID–Flu vaccine?<br>- Strongly agree;<br>- More agree than disagree;<br>- Neither agree nor disagree;<br>- More disagree than agree;<br>- Strongly disagree.	No	Yes

[a]This response option was available only in the 2021 survey
